# Functional Analysis of Zinc Finger Protein Transcription Factor *ZmZFP69* Under Low-Temperature Stress at Maize Seedling Stage

**DOI:** 10.3390/plants14142114

**Published:** 2025-07-09

**Authors:** Si-Nan Li, Yan Sun, Yun-Long Li, Ming-Hao Sun, Shu-Jun Li, Yue Yin, Tao Yu, Xin Li, Quan Cai, Jian-Guo Zhang

**Affiliations:** Maize Research Institute, Heilongjiang Academy of Agricultural Sciences, Harbin 150086, China; lee18686774002@126.com (S.-N.L.);

**Keywords:** zinc finger protein, *ZmZFP69*, maize, low-temperature stress

## Abstract

Maize (*Zea mays* L.) seedlings are highly susceptible to low-temperature stress, which significantly impacts maize yield and quality. A zinc finger protein transcription factor (*ZmZFP69*) mutant and a control (B73) maize inbred line were subjected to low-temperature treatment, and changes in the phenotypic characteristics, hormone levels, and other indicators before and after the treatment were systematically identified. Subsequently, a combined RNA-seq and DAP-seq analysis was conducted to explore the influence of ZmZFP69 on the promoters of downstream genes. Finally, the proteins interacting with ZmZFP69 were examined using InterProDesign combined with BiFC and subcellular localization. The *zmzfp69* homozygous mutant maize inbred line exhibited enhanced low-temperature tolerance compared to the control. RNA-seq and DAP-seq analyses revealed that *ZmZFP69* binds to the *ZmAOX2* gene promoter, significantly suppressing its expression. The interaction between ZmZFP69 and the downstream protein ZmBG6 was confirmed by InterProDesign, subcellular localization, and BiFC assays. *ZmZFP69* negatively regulates maize seedling low-temperature tolerance by inhibiting *ZmAOX2* expression and interacting with ZmBG6.

## 1. Introduction

Zinc finger proteins (ZFPs) are one of the largest categories of transcription factors in plants. As important regulatory proteins, they play key roles in multiple physiological processes such as plant growth and development and responses to environmental stress.

ZFPs present rich polymorphic characteristics in terms of their structure. According to the sequence of cysteine (Cys) and histidine (His) residues in its secondary structure, it can be classified into multiple subgroups. These include Cys2/His2 type (C2H2), C3H, C3HC4, C2HC5, C4HC3, C2HC, C4, C6, and C8 [1].

In the transcriptional regulatory system of plants, zinc finger proteins (ZFPs) have developed into a large and functionally critical family of transcription factors. As important regulatory elements in plants, they are widely involved in various physiological and biochemical processes and play an irreplaceable role in multiple functions such as plant growth and development and the stress response [2].

Low-temperature stress is an important environmental factor affecting plant growth and development, often leading to injury or death. When plants are exposed to low temperatures, their intracellular biological membranes become less fluid, damaging the membrane structure and subsequently affecting normal cell functions. At the same time, low temperatures can also inhibit various enzymes in plants, hindering physiological processes such as photosynthesis and respiration, and slowing down the growth rate. In addition, low-temperature stress may also cause the water inside cells to freeze, forming ice crystals. The formation of ice crystals can damage the cell structure, leading to rupture. In severe cases, it can cause the death of the plant [3,4].

The C2H2-type zinc finger protein can significantly enhance the cold tolerance of plants by directly regulating downstream cold-related genes. For example, *ZAT12* can effectively regulate plants’ cold adaptation process by precisely controlling the expression of 15 cold-inhibited genes and 9 cold-induced genes. In addition, *ZAT12* also has the ability to down-regulate *CBF* gene expression, which demonstrates that it plays a key negative regulatory role in the cold stress adaptation process in plants. Through this regulatory mechanism, it helps plants cope better with the challenges of cold environments and maintain their own physiological balance. *SCOF-1* can significantly enhance the activity of *SGBF-1* bound to the ABRE sequence, thereby promoting the expression of the *COR* gene and enhancing cold resistance [5]. *SCOF-1* has also demonstrated the ability to enhance cold tolerance in genetically modified sweet potatoes (*Ipomoea batatas* (L.) Lam.) [6]. *SlCZFP1* enhances the cold tolerance of transgenic Arabidopsis thaliana and rice (*Oryza sativa* L.) by inducing the continuous expression of *COR* or low-temperature response genes [7]. The low-temperature stress-related gene *COR6.6* was significantly up-regulated in *GmZF1* transgenic plants, indicating that *GmZF1* regulates the low-temperature stress resistance of transgenic Arabidopsis thaliana by binding to *COR6.6*’s promoter region [8]. In bananas (*Musa acuminata Colla*), *MaC2H2-2* and *MaC2H2-3* overexpression significantly inhibited the transcription of *MaICE1*, a key component of the cold signaling pathway. It can be seen from this that *MaC2H2s* is highly likely to enhance the cold resistance of bananas by inhibiting the process of *MaICE1* transcription [9].

Other C2H2-type zinc finger proteins also regulate the physiological responses of plants in a similar way through the ABA signaling pathway, synergistically enhancing plants’ resistance to low-temperature stress and enabling them to better adapt to cold environments [10].

ABA treatment can significantly induce *GmZF1* expression, a phenomenon suggesting that *GmZF1* is likely to be involved in the ABA-dependent signaling pathway. ABA, as an important signaling molecule for plants’ response to adverse conditions, plays a key role in the process of plants resisting adverse stress, such as low temperatures, through the signaling pathways it mediates. *GmZF1* expression induced by ABA indicates that it plays an important role in the ABA signal transduction process. It may enhance plants’ tolerance to adverse conditions by regulating the expression of downstream genes, providing new clues on plants’ stress resistance mechanisms [8]. However, the mechanism of ZFPs in the process of low-temperature stress in corn has not been reported.

In maize (*Zea mays* L.) ’s cold stress response, the IAA signaling pathway is closely related to tryptamine, tryptophan, melatonin, and other substances. Tryptophan is a key precursor for IAA biosynthesis. Tryptophan can be converted into indole-3-pyruvic acid (IPA) through the action of tryptophan aminotransferase, and then IPA is further converted into IAA by Yucca flavin-monooxygenase [11]. The synthesized IAA can regulate maize growth and development, such as through adjusting the root system architecture and stomatal development, so as to enhance the adaptability of maize to cold stress [12]. In addition, tryptophan can also affect the content of osmoregulatory substances in maize under cold stress conditions, such as through promoting proline synthesis, and playing a role in maintaining the cell’s osmotic balance and reducing damage caused by cold stress. Tryptamine is also a tryptophan metabolite. In the plant stress response process, tryptamine can be further synthesized into melatonin through a series of reactions. Although there have been no reports specifically on tryptamine’s direct participation in the IAA signaling pathway in maize under cold stress, as an important intermediate in melatonin synthesis, it may affect the IAA signaling pathway by regulating the melatonin content, so as to ensure the normal growth and development of maize under cold stress conditions, and may also participate in the cold stress response [13].

Due to the high geographical latitude of Heilongjiang Province, the annual accumulated temperature is lower than that of other provinces high-yield and stable-yield maize varieties are limited, and the maize production process is easily affected by the low temperature and cold damage, resulting in a decline in yield and quality.

In this study, the inbred lines of a *ZmZFP69* (*Zm00001d052815*) maize mutant and a control were used as experimental materials to verify the physiological manifestations of the mutant maize’s low-temperature tolerance at the seedling stage. Subsequently, multi-omics analysis methods were employed to elucidate the molecular mechanism of its low-temperature tolerance, providing a theoretical basis for breeding low-temperature-tolerant maize.

## 2. Results

### 2.1. Identification of Low-Temperature Resistance of Mutant Materials

#### 2.1.1. Phenotypic Identification of Mutant Materials

**Figure 1:**
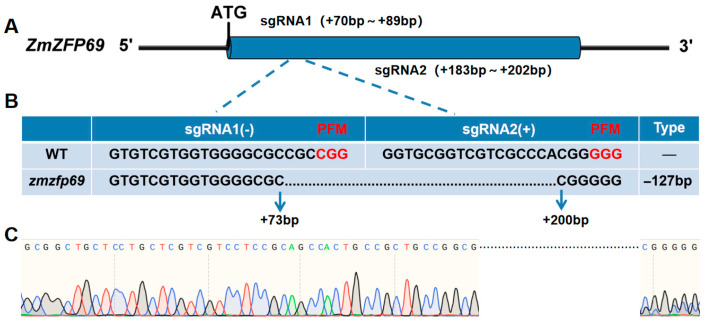
Identification of *zmzfp69* homozygous mutant materials: (**A**) Mutant gene sequence diagram. (**B**) *Zmzfp69* mutant gene editing type. (**C**) *Zmzfp69* mutant gene editing type sequencing profile.

The *zmzfp69* homozygous mutant material selected in this study was detected using Sanger sequencing. The results showed that compared with the wild-type material, this mutant had a 127-base deletion in the target gene sequence region, thereby leading to the premature termination of the amino acid sequence (Figure 1).

**Figure 2:**
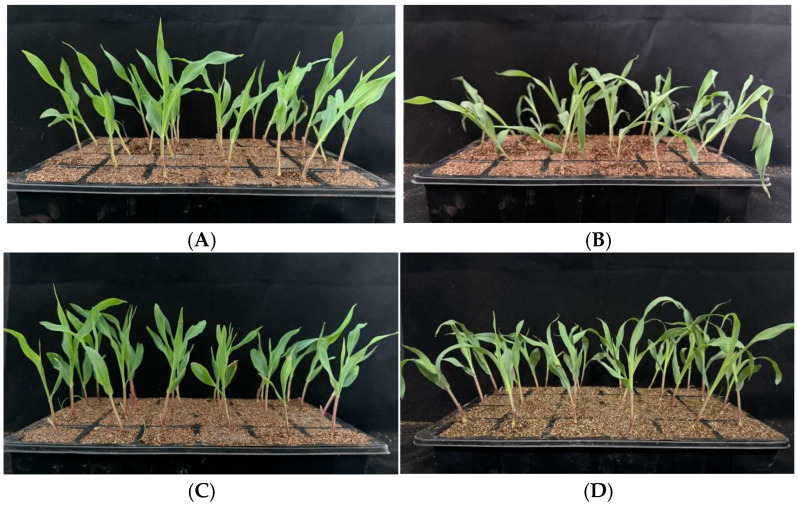
Identification of low-temperature resistance properties of the *zmzfp69* mutant materials. All of the test materials were maize inbred lines growing to the three-leaf stage (V2). Among them, (**A**) shows the phenotype of the wild-type material treated at 25 °C for 48 h, and (**B**) shows the phenotype of the wild-type material treated at 4 °C for 48 h. (**C**) shows the phenotype of the homozygous mutant material treated at 25 °C for 48 h, and (**D**) shows the phenotype of the homozygous mutant material treated at 4 °C for 48 h. All materials were restored in a 25 °C incubator for 24 h after low-temperature treatment.

**Figure 3:**
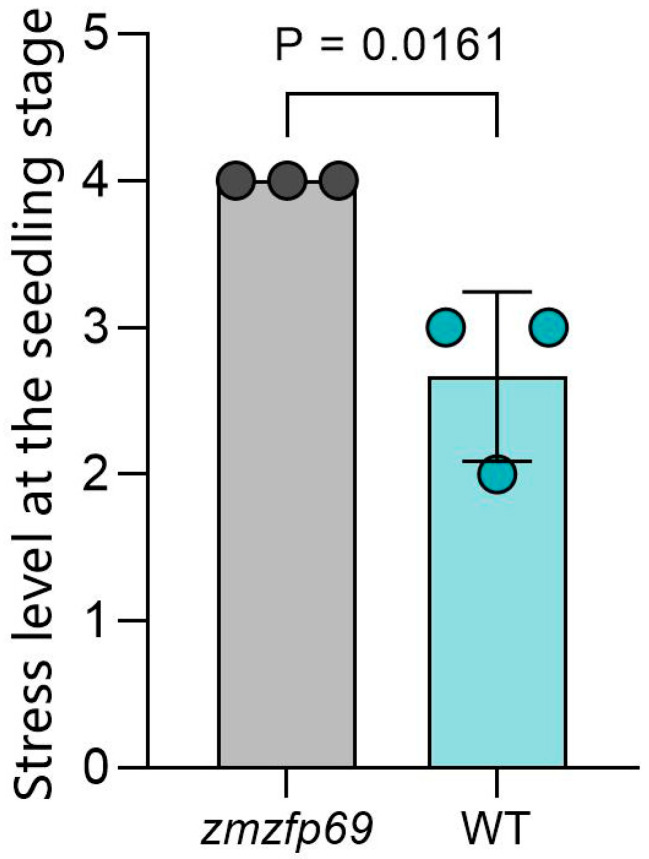
Stress levels at the seedling stage of maize inbred lines. *n* = 3; 50 seedlings per replicate.

**Figure 4:**
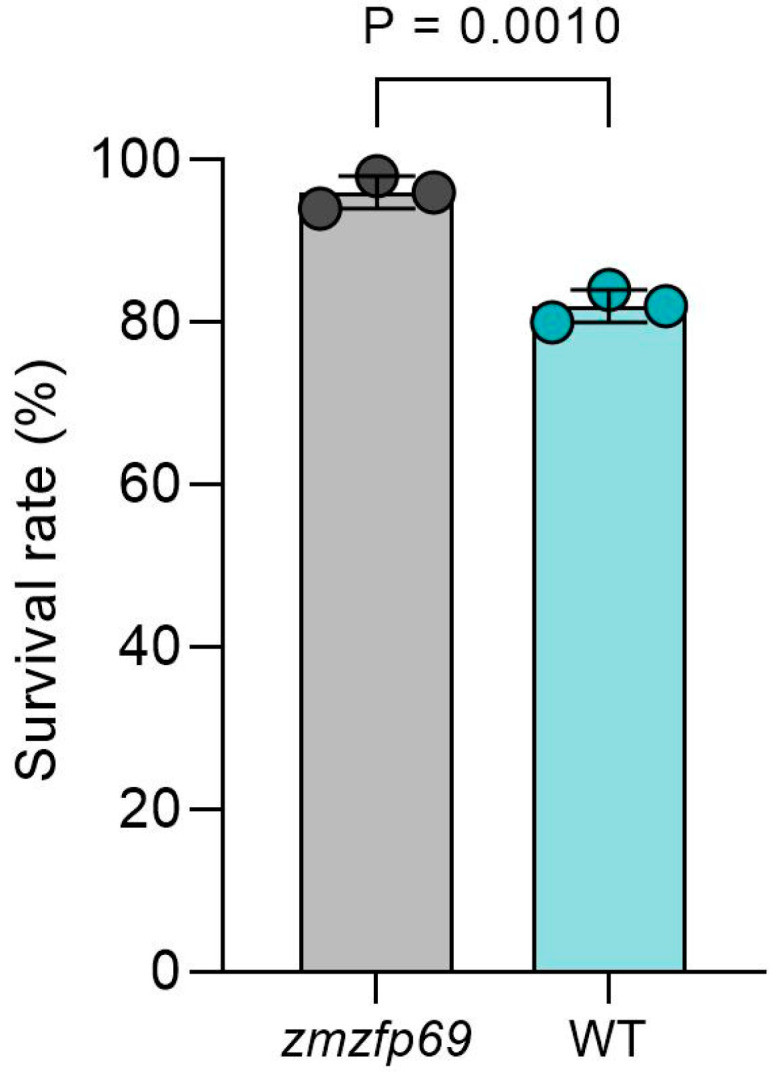
Survival rate of inbred lines after low-temperature stress (%). *n* = 3; 50 seedlings per replicate.

The low-temperature tolerance of the obtained mutant homozygous plants and control materials was measured (Figure 2). The results showed that compared with the control, the mutant materials exhibited less leaf atrophy and chlorosis, and the stress level and survival rate at the seedling stage were also higher (Figure 3 and Figure 4). The mutant displayed better low-temperature tolerance.

#### 2.1.2. Determination of Hormone Content in Mutant Materials

**Figure 5:**
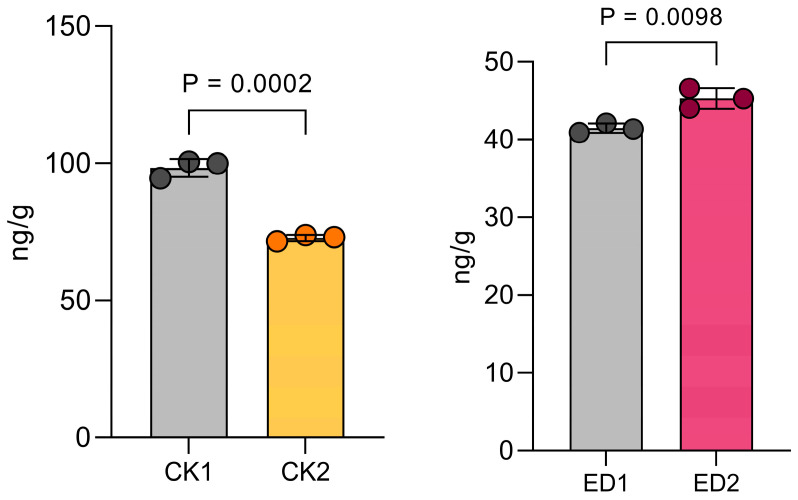
Tryptamine (TRA) content in WT and *zmzfp69* mutants under 4 °C treatment. CK1/CK2: WT before/after low-temperature treatment; ED1/ED2: *zmzfp69* mutant before/after treatment. *n* = 3 biological replicates, mean ± SD. *p* < 0.05, *p* < 0.01 (Student’s *t*-test).

The wild-type materials and mutant materials were subjected to low-temperature treatment in a 4 °C incubator. The treated leaves were taken for hormone content determination, and the results are shown in Figure 5. After the low-temperature treatment (ED2), the mutant material showed a significant increase in the tryptamine (TRA) content compared to that in its pre-treatment state (ED1). By contrast, the WT (CK2) exhibited lower TRA levels than the pre-treated WT (CK1). Given that ZmZFP69 is an auxin synthesis-related gene, and combined with this phenotype, it can be assumed that this gene inhibits TRA synthesis. After the knockout of this gene, the TRA synthesis pathway is derepressed, affecting auxin synthesis and resulting in a significant increase in the low-temperature resistance of the mutant material.

### 2.2. ZmZFP69 Downstream Target Gene Screening Through Multi-Omics Joint Analysis

#### 2.2.1. *ZmZFP69* Potential Downstream Target Gene Screening by RNA-Seq

In order to screen the potential downstream target genes of *ZmZFP69*, the mutant material (*zmzfp69*) and the control material (CK) were treated at a low temperature of 4 °C during the trilateral stage. The sampling time was 0/24 h, and transcriptome sequencing was repeated three times for each sample. After sequencing quality control (QC), joint elimination, raw read filtering, and low-copy sequence removal, a total of 295,953,316 reads were obtained. The Q20 values of all sequencing raw data were above 97%, the Q30 values were above 93%, and the G/C base contents were above 54.34%. It can be concluded from this that the base quality of the transcriptome data from the *ZmZFP69* and CK leaf materials under low-temperature stress for 24 and 48 h during the trilobar stage is relatively good (Table S1).

**Figure 6:**
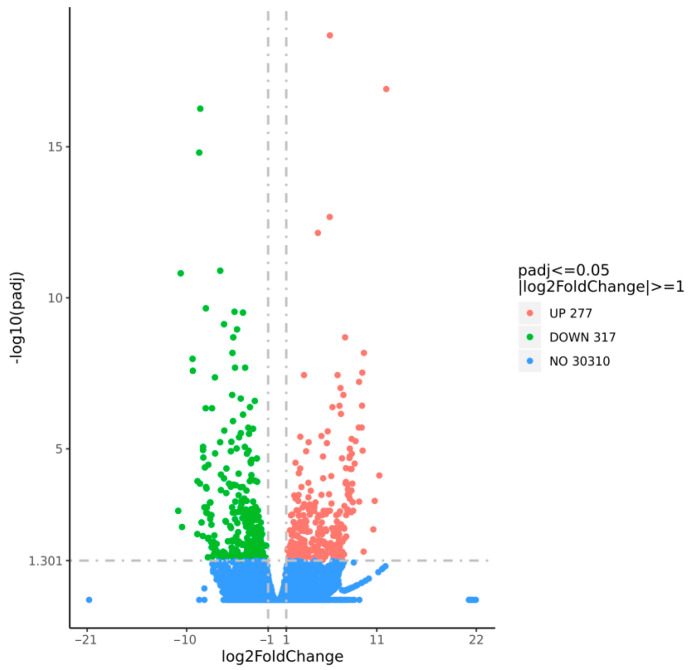
Volcano plot of differentially expressed genes (DEGs) in *zmzfp69* vs. WT under 4 °C treatment for 24 h. DEGs were identified with |log2FC| ≥ 1 and adj. *p* ≤ 0.05 (*n* = 3 biological replicates).

The transcriptome data of the leaves subjected to low-temperature stress during the seedling stage of the corn and CK leaf samples were compared and analyzed to explore the potential downstream target genes of *ZmZFP69*. Under the screening criterion of fold change ≥ 0.5, an analysis of the differentially expressed genes was conducted. Compared with CK, there were a total of 594 differentially expressed genes in *ZmZFP69*, among which 277 genes showed up-regulated expression levels and 317 genes showed down-regulated expression levels (Figure 6).

**Figure 7:**
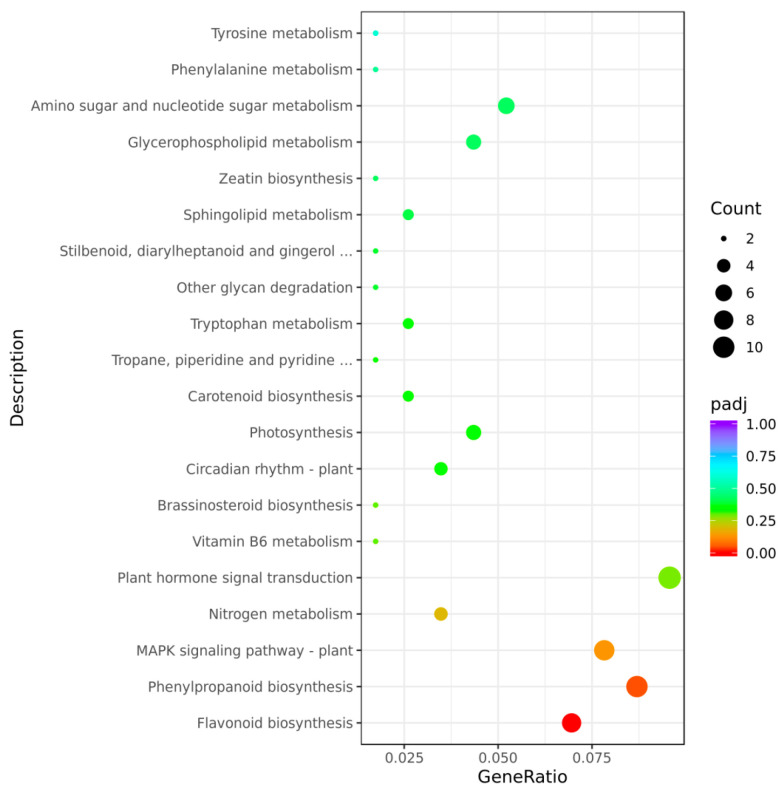
KEGG enrichment of DEGs from RNA-seq analysis of zmzfp69 and WT leaves under 4 °C treatment (24 h). Pathways with adj. *p* ≤ 0.05 are shown (*n* = 3 biological replicates).

After conducting a KEGG enrichment analysis on the differentially expressed genes, the results showed that these genes were enriched in a total of 77 pathways. Comprehensively considering the *p*-adjusted value and the number of genes enriched in each pathway, the analysis revealed that the differentially expressed genes were significantly enriched in plant hormone signal transduction, and this pathway plays a key regulatory role in the growth, development, and response to plants’ environmental stress: (1) The metabolites of tryptophan, as an essential amino acid, are engaged in plants’ growth regulation and defense responses. Tryptophan maintains the balance of ions and hormones within plants and enhances their cell membrane stability, thereby improving their abiotic stress tolerance to a certain extent [14]. (2) Phenylpropanoid biosynthesis, with its products (e.g., flavonoids) being crucial for cell wall formation and antioxidant capacity, was also enriched with DEGs. Flavonoids can be continuously synthesized and accumulate when plants encounter more extreme or persistent stresses. Some of them are deeply integrated into the lipid bilayer to protect membrane lipids and proteins from oxidative damage [15]. It is worth noting that the above enrichment results show similarity to the results of *ZmZFP69* mutant hormone determination (Figure 7).

#### 2.2.2. DNA Affinity Purification Sequencing (DAP-Seq)

The DAP-seq analysis of *ZmZFP69* was performed using the wheat germ protein expression system to screen the downstream target genes of *ZmZFP69* during the low-temperature stress process in maize seedlings. It can be seen from Table S2 that a total of 40,860,590 original data points were obtained in the IP group. After filtering the original data, a total of 40,625,660 clean reads were obtained. The Q20 value of the sequencing data of the IP group was about 98.72%, and the Q30 values were all above 95.55% (Table S2).

Following the DAP-seq analysis, a total of 8894 peaks were identified in the promoter regions (defined as 2 kb upstream of the transcription start site, TSS) (Table S3). To associate these peaks with potential target genes, we mapped each peak to the nearest gene within the promoter region. This mapping process considered the genomic coordinates of maize (Zmays_RefGen_V4), resulting in the annotation of 7091 genes that harbored promoter-bound peaks. Discrepancies between the peak count (8894) and gene count (7091) arise because multiple peaks can map to the promoter of a single gene, or a single peak may span multiple regulatory elements within a promoter.

**Figure 8:**
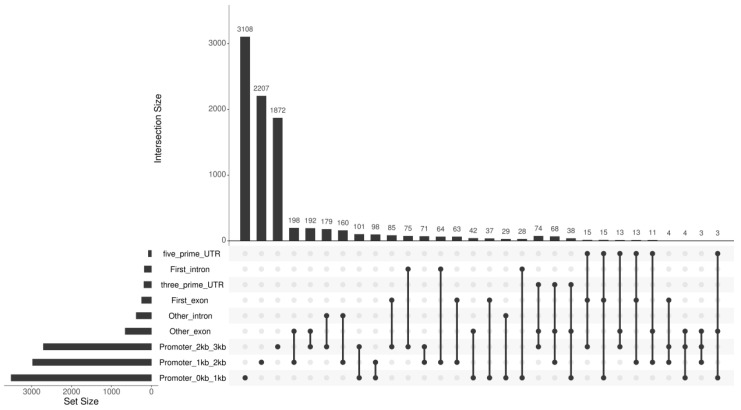
Schematic diagram of the genetic location of peaks mined by DAP-seq. Peaks were annotated based on their distance from the transcription start site (TSS); total promoter-associated peaks: 8894, corresponding to 7091 genes.

For clarity, Figure 8 illustrates the genomic distribution of the peaks, with the majority (64.6%) located within 1 kb of the TSS (Promoter_0 kb_1 kb), followed by 23.5% in the 1–2 kb region (Promoter_1 kb_2 kb) and 11.9% in the 2–3 kb region (Promoter_2 kb_3 kb). The remaining peaks were distributed across other genomic regions (e.g., exons or introns). This annotation strategy ensures that we focus on the genes directly regulated by *ZmZFP69* binding in their proximal promoter regions. The total length of the peaks was 2,851,132 bp. The average length of the peaks was 320.57 bp, and the average read coverage reached 15.31.

#### 2.2.3. Combined Analysis of DAP-Seq and RNA-Seq to Screen *ZmZFP69* Target Genes

In order to explore the potential downstream target genes of *ZmZFP69* during the low-temperature stress process of maize seedlings, the RNA-seq and DAP-seq data were jointly analyzed. Combined with the results of the determination of physiological indicators and hormone contents of the *ZmZFP69* mutant during low-temperature stress in the maize seedlings, the 594 differentially expressed genes involved in plant hormone signal transduction screened by the RNA-seq analysis were mainly analyzed in combination with the 7091 genes identified using the DAP-seq peaks.

Using the 7091 genes annotated according to the DAP-seq promoter peaks (covering key regulatory regions like the 0–3 kb upstream of the TSS, as visualized in genomic distribution analyses), we performed a strict overlap analysis with the 594 hormone-signaling-related DEGs identified from the RNA-seq analysis. This dual-filtering strategy ensured that only the genes directly bound by *ZmZFP69* (via DAP-seq) and transcriptionally responsive to cold stress (via RNA-seq) were retained.

**Figure 9:**
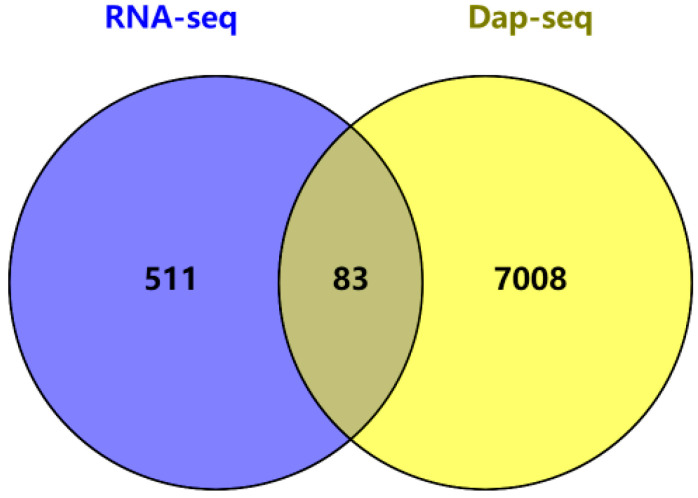
Venn diagram of the combined analysis of RNA-seq and DAP-seq data.

As can be seen from Figure 9 and Table S4, the combined analysis of RNA-seq and DAP-seq identified 83 genes. They, respectively, include the plant hormone signal transduction transcription factors *ZmPIF4* and *ZmZH3*, the plant MAPK signal response gene *ZmPYL2*, and the tryptophan metabolism-related gene *ZmAOX2* in the important tryptophan (TRA) synthesis pathway (Table 1). *ZmAOX2* (indoleacetaldehyde oxidase) is involved in tryptamine (TRA) synthesis via the tryptophan metabolism pathway. Specifically, it catalyzes the oxidation of indoleacetaldehyde to indoleacetic acid (IAA), with tryptamine serving as an intermediate precursor. Based on the determination of the hormone content in the *ZmZFP69* mutant, *ZmAOX2* was selected as the key target gene of *ZmZFP69* for verification.

### 2.3. Luciferase Assay Verified That ZmZFP69 Recognizes the ZmAOX2 Promoter

**Figure 10:**
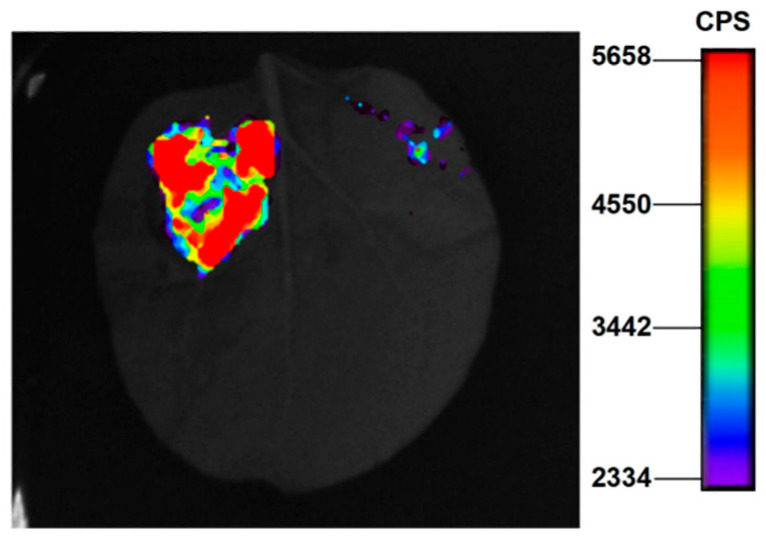
Luciferase imaging of *N. benthamiana* leaves co-transfected with *ZmAOX2* promoter-LUC and *ZmZFP69*-Flag vectors. The left side of the leaf in the figure is the control group, and the vector used is Ev+800-35S-*ZmAOX2*-luc. On the right is the experimental group, using the vector *ZmZFP69*-flag+800-35S-*ZmAOX2*-luc.

**Figure 11:**
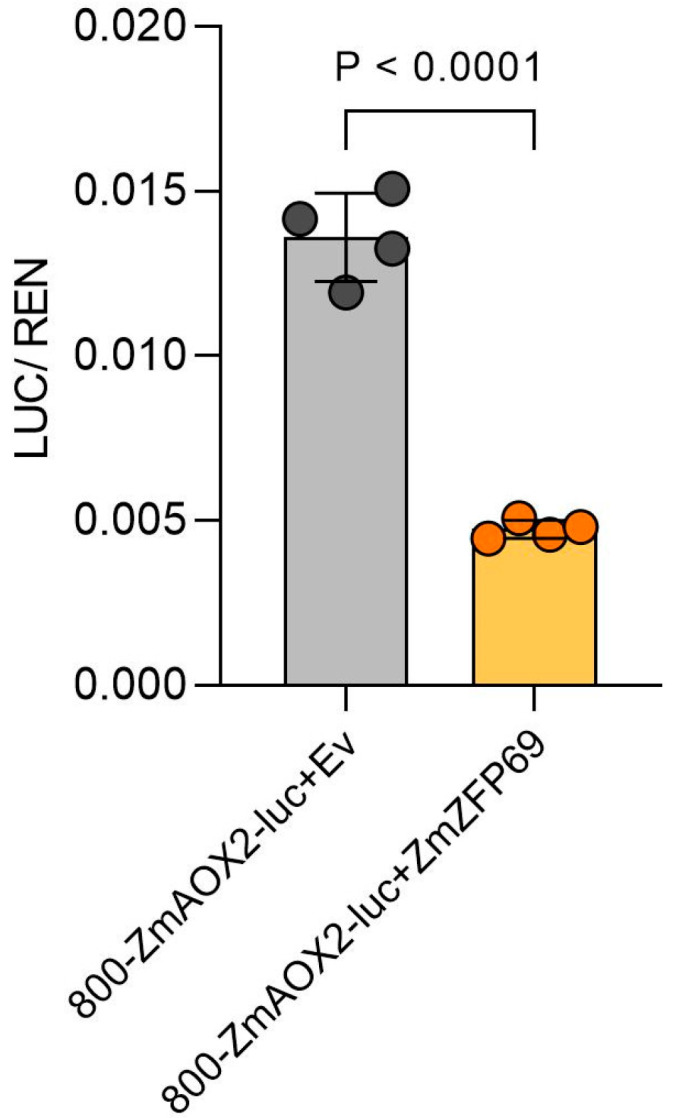
Dual bioluminescence LUC/REN ratio assay. The statistical significance of differences between the experimental group and the control group was analyzed using Student’s *t*-test, with *p* < 0.0001 indicating an extremely significant difference.

Whether *ZmZFP69* recognizes the *ZmAOX2* promoter was detected using the tobacco luciferase imaging assay. It can be seen in Figure 10 that bioluminescence was detected in the control group (on the left, pGreenII 800-*ZmAOX2*-35smini-Luc+pCambia1300-221-Flag), while in the experimental group (on the right), the bioluminescence value could not be detected by pGreenII 800-*ZmAOX2*-35smini-Luc+pCambia1300-221-Flag-ZmZFP69. It can be seen in Figure 11 that the LUC/REN ratio in the experimental group was significantly lower than that in the control group. Based on the determination and analysis of the fluorescence values of LUC and REN, it was found that *ZmZFP69* could significantly inhibit *ZmAOX2* expression.

### 2.4. Identification of Downstream Interacting Proteins with InterProDesign

By using the RFdiffusion tool to design bait proteins for the designed *ZmZFP69* domain, the top 100 proteins with a FoldSeek alignment score (Bit score) greater than 50 were extracted for the calculation of the Hdock binding energy and the prediction of the AF3 (AlphaFold3) complex. Table 2 lists the predicted interaction proteins. Complexes with |HDock score| ≥ 300 were selected for validation.

**Figure 12:**
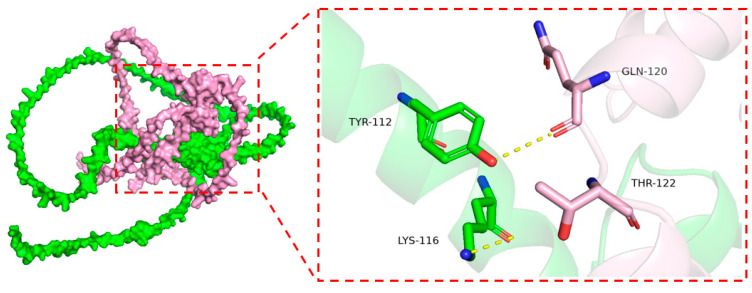
Three-dimensional binding model diagram of ZmZFP69 (green) and ZmBG6 (pink).

Meanwhile, based on the results of the InterProDesign analysis, we studied the binding modes and molecular interactions between the target proteins using molecular docking to explore their potential biological relevance and structural compatibility. As shown in Figure 12, the overall structures of the proteins were visualized using a cartoon representation, which highlights the folding and domain organization, while the key amino acid residues involved in the interaction interface were displayed as sticks to emphasize their direct participation in binding. The docking analysis revealed that the target proteins possess favorable binding energies, indicating a stable interaction. Notably, GLN-120 and THR-122 of Chain ZmBG6 can form two hydrogen bonds with TYR-112 and LYS-116, respectively, on Chain ZmZFP69. It has been proven that these residues interact.

### 2.5. Subcellular Localization

**Figure 13:**
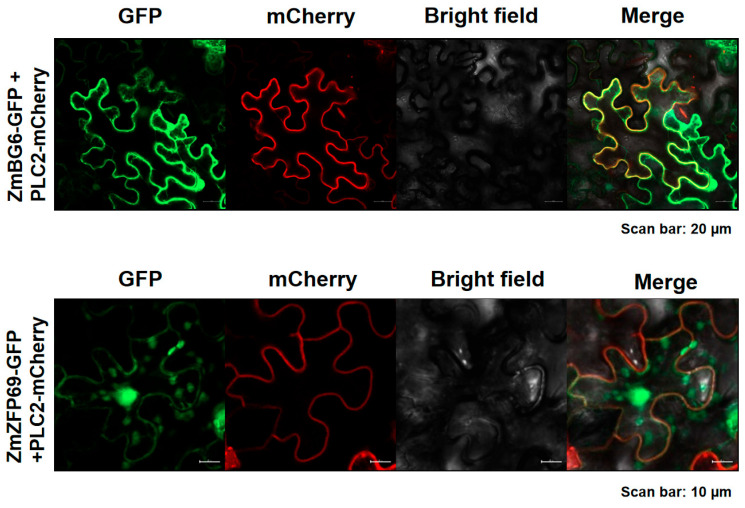
Subcellular localization of ZmZFP69 and ZmBG6.

Based on the constructed vector and membrane Marker-PCL2-MCherry, the localization of the ZmZFP69 and ZmBG6 proteins in plant cells was verified. The results showed that ZmBG6 and membrane marker PLC2-MCherry could be co-localized. ZmZFP69 can be co-located with the membrane marker PLC2-MCherry. Additionally, ZmZFP69 has a strong localization signal (Figure 13).

### 2.6. BiFC Verification of Interacting Proteins

**Figure 14:**
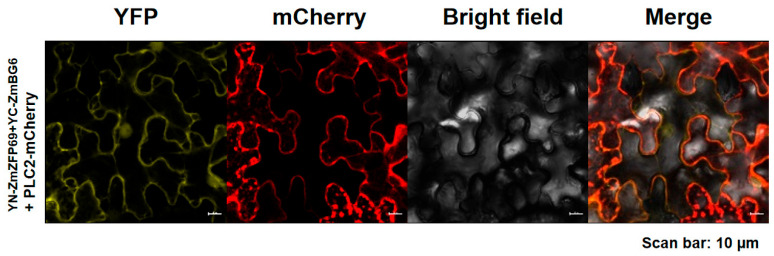
BiFC verifies the interaction between ZmZFP69 and ZmBG6.

In order to verify whether ZmZFP69 and ZmBG6 proteins interact in plants, pEarleyGate201-YN201-ZmZFP69 + pEarleyGate202-YC202-ZmBG6 were jointly used to infect the leaves of *N. benthamiana*. The yellow fluorescence signal of YFP was observed in the tobacco leaf cells after injection under a laser confocal microscope. The experimental results are shown in Figure 14. Yellow fluorescence signals appeared in the cells of the *N. benthamiana* leaves in the experimental group. Also, these signals could be co-located with the membrane marker PLC2-mcherry (Figure 14). The verification of the bimolecular fluorescence complementarity technique indicates that there is a direct interaction between ZmZFP69 and ZmBG6 in plants.

## 3. Discussion

When plants are subjected to abiotic stress, a complex and precise transcriptional regulatory network system is constructed within them. In this network, transcription factors play a core hub role. By precisely regulating the expression levels of downstream target genes, they mediate the responses of plants to various stresses and facilitate their adaptation to adverse environments [16]. Data from numerous studies have shown that zinc finger protein transcription factors are of great significance in plants’ resistance to abiotic stresses such as drought, salinity, and low temperatures [7,17,18]. Such transcription factors can participate in plants’ response and adaptation to stress by regulating the expression of downstream genes, and can help plants maintain normal functioning in harsh environments [19,20,21].

*Di19*, as a transcriptional activator, can directly promote the expression of the disease-related genes *PR1*, *PR2*, and *PR5*, thereby enhancing the drought resistance of Arabidopsis thaliana [22]. *ZAT6* enhances resistance to salt stress, drought stress, and pathogenic bacteria by directly activating the expression of the stress response gene *CBF* and pathogenic bacterial infection-related genes [23]. In addition, both *ZAT7* and *STZ* have the ability to enhance plants’ salt tolerance. When dealing with drought stress, *ZAT7*, *STZ*, and *ZAT18* all play positive regulatory roles [17,18,24]. For soybeans (*Glycine max* (L.) *Merr.*), plants that overexpress the zinc finger transcription factor *SCOF-1* can enhance their cold tolerance by increasing the expression levels of the cold regulation genes *COR15a*, *COR47*, and *RD29B*. Further studies have found that *SCOF-1* can interact with the bZIP transcription factor *SGBF-1*. This interaction enhances the binding of *SGBF-1* to ABRE [10], thereby further strengthening cold tolerance [10]. In addition, *ZAT6* can directly activate the expression of *GSH1*, a gene related to the chelating hormone (PC) synthesis pathway, thereby enhancing the tolerance of plants to cadmium [25]. *ZAT11* is involved in the process of programmed cell death induced by oxidative stress and plays a negative regulatory role in nickel ion resistance [26,27].

The molecular mechanisms revealed in this study show obvious conservation and similarities with the findings of related studies on other species. From the perspective of transcriptional regulation, the mechanism of *ZmZFP69*′s inhibition of *ZmAOX2* is similar to how *OsZOS2-19* in rice regulates cold resistance by inhibiting *OsPGL12* and *OsWRKY71* [28]. In addition, this mechanism is also in line with the pattern seen in Chinese cabbage (*Brassica rapa* L. ssp. *pekinensis*), in which *BcZAT12* directly binds to the *BcCBF1* promoter and inhibits its transcription [29]. Furthermore, the mode of action of *ZmZFP69* is also similar to the mode in which the *COOL1* gene in maize negatively regulates cold tolerance by inhibiting the DREB1/CBF pathway [30]. This conservation between different species indicates that the C2H2-type zinc finger protein may have formed a core regulatory module of “transcriptional inhibition” by targeting the promoter region of stress response genes.

In terms of gene discovery strategies, a systematic approach of “phenotypic screening–omics enrichment–functional verification” was adopted to precisely locate key gene families. This research path is similar to those used to find the rice cold tolerance gene *OsZOS2-19* [28] and the maize *COOL1* gene [30], fully demonstrating the core value of multi-omics technology in analyzing complex traits. In the molecular mechanism dimension, the negative regulatory characteristics of *ZmZFP69* share commonalities with the mechanism by which *BcZAT12* in Chinese cabbage without head formation inhibits the CBF pathway to regulate cold tolerance [5], and through which the STZ protein of Arabidopsis thaliana negatively regulates the cold response by inhibiting *COR* gene expression [17], indicating that the C2H2-type zinc finger protein is present in different plant species, participating in the low-temperature stress response process through similar regulatory pathways. Furthermore, the dual regulatory patterns of the “interaction between transcription factors and target genes” and “interaction between proteins” revealed in this study complement the complex regulatory mechanism of rice *OsZOS2-19* [28], greatly expanding our understanding of the functional diversity of zinc finger proteins.

Previous studies on the interaction between low temperature and auxin signals have shown that a low-temperature environment can reduce the auxin content in apples (*Malus domestica Borkh.*) [31]. However, a different situation has been seen in rice. Low temperatures can promote an increase in the auxin content and also up-regulate the expression of some auxin signaling genes [32]. The *GH3* gene, encoding auxin conjugates, can maintain normal auxin levels in plants by combining auxin with certain amino acids [33,34]. A study by Park et al. demonstrated that a *GH3* gene (*wes1-D*) mutant with a gain-of-function mutation in Arabidopsis thaliana exhibited a significant frost-resistant phenotype, and the expression levels of *CBF* and *RD29A* genes in the mutant plants were significantly higher than those in the wild type [35]. In addition, studies have found that low temperatures can have an impact on the gravitropism of roots [36]. Through investigating cell biology, researchers have observed that under low-temperature conditions, the transport of auxin output carriers *PIN2* and *PIN3* within cells changes. This change affects the auxin transport process and eventually leads to alterations in the gravitropism of roots [36].

Through transcriptomic analysis, it can be seen that in overexpressing plants, some auxin response genes generally show down-regulated expression, and both *AZF1* and *AZF2* can bind to the promoters of the auxin response genes *SAUR20* and *SAUR63*, thereby inhibiting their expression [37]. Tryptamine is a key intermediate in melatonin (MET) synthesis in plants and plays an important role in their cold stress response. In plants, tryptamine is formed through the decarboxylation of tryptophan, catalyzed by tryptophan decarboxylase (TDC), and is one of the precursor substances for melatonin synthesis. Cold stress can induce an increase in the melatonin content in plants. As an intermediate in melatonin synthesis, tryptamine synthesis and metabolism may be regulated by cold stress. For example, under cold stress, the activity of TDC in plants may increase, promoting the conversion of tryptophan to tryptamine, thus increasing melatonin synthesis and enhancing the tolerance of plants to cold stress [38].

This study found that *ZmZFP69* negatively regulates cold tolerance by inhibiting *ZmAOX2* expression, thereby reducing auxin precursor synthesis. This is supported by the decreased TRY levels in the WT vs. mutants (Figure 5). *ZmZFP69* maintains the stability of the intracellular environment by regulating the auxin metabolic pathway, providing a brand-new empirical basis for the theoretical mechanism of “plant stress resistance and growth trade-off”. In addition, the research successfully identified the function of “down-regulated negative regulatory factors” in the *C2H2* gene family in the response to cold stress, deviating from the traditional model in which “stress resistance genes are mostly activated through the up-regulation of expression”, and significantly broadening the range of genes to screen for their response to low-temperature stress from a research perspective.

The analysis of molecular mechanisms in this study reveals that *ZmZFP69* mainly exerts its function through two core pathways: Firstly, ZmZFP69 can directly bind to the promoter of the *ZmAOX2* (indole acetaldehyde oxidase) gene, thereby inhibiting its expression and ultimately reducing auxin precursor synthesis. Secondly, the ZmZFP69 protein interacts with the molecular chaperone regulatory protein ZmBG6, possibly by regulating the activity of molecular chaperones such as HSP70 to maintain protein homeostasis. This dual regulatory network operates collaboratively at the transcriptional level and the protein level, respectively, jointly constituting the molecular mechanism by which *ZmZFP69* negatively regulates the low-temperature stress response.

Meanwhile, according to the research results of Sun et al. (Mol Plant, 2021), the plant endoplasmic reticulum, chloroplasts, and mitochondria are the key organelles for protein synthesis, photosynthesis, metabolism, and energy generation. The maintenance and regulation of protein homeostasis in these organelles play a key role in the growth, development, and stress resistance of plants [39,40]. In this study, under low-temperature stress conditions, the significance of protein stability regulation is consistent with the mechanism by which the ZmZFP69 protein maintains protein homeostasis through interaction with a molecular chaperone. This further confirms the theoretical model that “protein homeostasis regulation is the core strategy for plant stress resistance”.

From the perspective of the mode of action, ZmZFP69 presents a dual regulatory role of “transcriptional inhibition + protein–protein interaction”, which echoes the complex mechanism of “transcriptional regulation + protein–protein interaction” of *OsZOS2-19* in rice. Furthermore, the interaction between ZmZFP69 and the molecular chaperone regulatory protein ZmBG6 of the BAG family is similar to the mechanism by which *SlBAGs* in tomatoes (*Solanum lycopersicum* L.) respond to abiotic stress by regulating the activity of HSP70 [41,42]. This fully demonstrates that the zinc finger protein can enhance the accuracy of plants’ responses to stress through multi-dimensional regulatory methods. Furthermore, studies have reported that *ZAT12* in Arabidopsis thaliana interacts with HSP90, thereby enhancing the plant’s heat tolerance [5], which further confirms the universality of the synergistic effect of transcription factors and molecular chaperones.

## 4. Materials and Methods

### 4.1. Identification of Low-Temperature Resistance Properties of Mutant Materials

#### 4.1.1. Phenotypic Identification of Mutant Materials

In this study, the maize inbred line materials were sown in seed culture boxes and then transferred to a light incubator for cultivation. The environmental parameters set inside the incubator are as follows: a constant temperature of 25 °C, and a photoperiod following an alternating pattern of 16 h of light and 8 h of darkness. When the seedlings grew to the three-leaf (V2) stage, the plants in the treatment group were subjected to low-temperature stress treatment: they were placed in a light incubator at 4 °C for continuous stress for 48 h, and then taken out and restored to growth conditions for 24 h under conditions of 25 °C and 12 h of light/12 h of darkness. The plants in the control group were maintained to grow normally in a light incubator at 25 °C for the duration of the experiment. Three biological replicates were set up, each of which included 50 seedling samples. After the experiment, the survival rate of seedlings in each repeat group and the injury level caused by low-temperature stress were statistically analyzed, and the average value of the three replicates was taken as the final statistical result.

Survival rate calculation formula:

Survival rate (%) = (Number of surviving seedlings/Total number of treated seeds) × 100%

Classification standards for low-temperature stress injuries:

LEVEL 5: There is no wilting of the plant leaves, and the overall growth state is normal.

LEVEL 4: Approximately 20% of the plants show slight wilting, and the edges of the affected leaves exhibit atrophy symptoms.

LEVEL 3: Approximately 50% of the plant leaves are suffering from low-temperature damage, but the heart leaves remain normal, and traces of low-temperature cold damage can be seen on the petioles and stems.

Level 2: Around 70% of the plant leaves are atrophied due to water loss, and the heart leaves have suffered mild cold damage.

LEVEL 1: Most leaves and heart leaves are damaged by frost, and 85% of the plants have died.

LEVEL 0: All leaves and heart leaves are affected by low-temperature cold damage, and the plant mortality rate has reached 100%.

In the survival rate and stress level trials, data are from 3 biological replicates (*n* = 3), each with 50 seedlings.

#### 4.1.2. Determination of Hormone Indicators of Maize Mutant Materials

(1) Chemicals and Reagents

HPLC-grade acetonitrile (ACN) and methanol (MeOH) were purchased from Merck (Darmstadt, Germany). MilliQ water (Millipore, Burlington, MA, USA) was used in all the experiments. All of the standards were purchased from Olchemim Ltd. (Olomouc, Czech Republic) and isoReag (Shanghai, China). Acetic acid and formic acid were bought from Sigma-Aldrich (St Louis, MO, USA). The stock solutions of standards were prepared at a concentration of 1 mg/mL in MeOH. All the stock solutions were stored at −20 °C. The stock solutions were diluted with MeOH to make working solutions before analysis.

(2) Sample Preparation and Extraction

A fresh plant sample was harvested, immediately frozen in liquid nitrogen, ground into powder (30 Hz, 1 min), and stored at −80 °C until needed. A mass of 50 mg of plant sample was weighed into a 2 mL plastic microtube and frozen in liquid nitrogen, dissolved in 1 mL methanol/water/formic acid (15:4:1, *v*/*v*/*v*). A volume of 10 μL of internal standard mixed solution (100 ng/mL) was added to the extract as an internal standard (IS) for the quantification. The mixture was vortexed for 10 min and then centrifuged for 5 min (12,000 r/min, and 4 °C); the supernatant was transferred to clean plastic microtubes, followed by evaporation to dryness, and was dissolved in 100 μL of 80% methanol (*v*/*v*) and filtered through a 0.22 μm membrane filter for further LC-MS/MS analysis.

(3) UPLC Conditions

The sample extracts were analyzed using a UPLC-ESI-MS/MS system (UPLC, ExionLC™ AD, https://sciex.com.cn/; MS, QTRAP^®^ 6500+, https://sciex.com.cn/). The analytical conditions were as follows: LC—column, Waters ACQUITY UPLC HSS T3 C18 (100 mm × 2.1 mm i.d., 1.8 µm); solvent system, water with 0.04% acetic acid (A) and acetonitrile with 0.04% acetic acid (B); gradient program, started at 5% B (0–1 min), increased to 95% B (1–8 min), 95% B (8–9 min), and finally ramped back up to 5% B (9.1–12 min); flow rate, 0.35 mL/min; temperature, 40 °C; injection volume: 2 μL.

(4) ESI-MS/MS Conditions

Linear ion trap (LIT) and triple quadrupole (QQQ) scans were acquired on a triple quadrupole–linear ion trap mass spectrometer (QTRAP), QTRAP^®^ 6500+ LC-MS/MS System, equipped with an ESI Turbo Ion-Spray interface, operating in both the positive and negative ion modes and controlled by the Analyst 1.6.3 software (Sciex, Spokane, WA, USA). The ESI source operation parameters were as follows: ion source, ESI+/−; source temperature, 550 °C; ion spray voltage (IS), 5500 V (Positive), −4500 V (Negative); curtain gas (CUR) was set at 35 psi. Phytohormones were analyzed using scheduled multiple reaction monitoring (MRM). Data acquisitions were performed using the Analyst 1.6.3 software (Sciex). Multiquant 3.0.3 software (Sciex) was used to quantify all the metabolites. The mass spectrometer parameters, including the declustering potentials (DPs) and collision energies (CEs) for individual MRM transitions, were set with further DP and CE optimization. A specific set of MRM transitions was monitored for each period according to the metabolites eluted within this period.

### 4.2. Multi-Omics Joint Analysis

#### 4.2.1. RNA-Seq

The leaves at the three-leaf stage of the mutant (zmzfp69) and control (B73) maize inbred lines were treated at low temperature for 0 h, 24 h, and 48 h as the experimental materials. RNA integrity was assessed using the Bioanalyzer 2100 system (Agilent Technologies, Palo Alto, CA, USA) after extraction from leaves. Messenger RNA was purified from total RNA using poly-T oligo-attached magnetic beads. After fragmentation, the first strand of cDNA was synthesized using random hexamer primers. Then, the second strand of cDNA was synthesized using dUTP instead of dTTP. The directional library was ready after end repair, A-tailing, adapter ligation, size selection, USER enzyme digestion, amplification, and purification. The library was checked with Qubit and real-time PCR for quantification and with a bioanalyzer for size distribution detection. After library quality control, different libraries were pooled based on the effective concentration and targeted data amount, and then subjected to Illumina sequencing. Raw data (raw reads) of fastq format were first processed through the fastp software (0.24.1). The Q20, Q30, and GC content of the clean data were calculated. All the downstream analyses were based on clean data of high quality. Reference genome and gene model annotation files were downloaded from the genome website directly (https://phytozome-next.jgi.doe.gov/info/Zmays_RefGen_V4, accessed on 20 November 2024). An index of the reference genome was built using Hisat2 v2.0.5, and paired-end clean reads were aligned to the reference genome using Hisat2 v2.0.5. The mapped reads of each sample were assembled by String Tie (v1.3.3b) using a reference-based approach. Feature Counts v1.5.0-p3 was used to count the read numbers mapped to each gene. And then, FPKM of each gene was calculated based on the length of the gene and read counts mapped to genes. Differential expression analysis for two conditions/groups was performed using the DESeq2 R package (1.20.0). DESeq2 provides statistical programs for determining differential expression in digital gene expression data using models based on the negative binomial distribution. The resulting *p*-value is adjusted using Benjamini and Hochberg’s methods to control the error discovery rate. The corrected *p*-value ≤ 0.05 and |log2^(fold change)^| ≥ 1 were set as the threshold of significant differential expression methods to control the error discovery rate. Gene Ontology (GO) enrichment analysis of differentially expressed genes was implemented using the cluster Profiler R package, in which gene length bias was corrected. GO terms with corrected *p*-values less than 0.05 were considered significantly enriched according to differentially expressed genes. We used the cluster Profiler R package to test the statistical enrichment of differentially expressed genes in KEGG pathways. RNA-seq was performed by Beijing Novogene Technology Co., Ltd. (Beijing, China) (https://www.novogene.cn/).

#### 4.2.2. DAP-Seq and Analysis

DAP-seq was performed according to a method previously described [43] by Wuhan IGENEBOOK Biotechnology Co., Ltd. (Wuhan, China) (http://www.igenebook.com). First, a DAP-seq genomic DNA (gDNA) library was prepared by attaching a short DNA sequencing adaptor onto purified and fragmented gDNA. The DAP gDNA library was prepared using a kit from NEBNext^®^ DNA Library Prep Master Mix Set for Illumina^®^ (NEB, #E6040S/L, Ipswich, MA, USA). *ZmZFP69* was fused to the HaloTag using a kit from pFN19K HaloTag T7 SP6 Flexi Vecto (Promega #G184A, Madison, WI, USA). *ZmZFP69* fused to HaloTag was expressed using a TnT SP6 High-Yield Wheat Germ Protein Expression System (L3260, Promega, Madison, WI, USA). The Magne HaloTag Beads and *ZmZFP69*-HaloTag mixture were incubated with 500 ng DNA library in 40 µL PBS buffer with slow rotation for 1 h at room temperature. The beads were washed five times with 200 µL PBS + NP40 (0.005%), resuspended in PBS buffer, the supernatant was removed, and 25 µL EB buffer was added and incubated for 10 min at 98 °C to elute the bound DNA from the beads. The correct DAP-seq library concentration to achieve a specific read count was calculated based on the library fragment size. The input was a directly obtained genome-wide DNA library. The correct DAP-seq library and input libraries were sequenced using an Illumina NovaSeq 6000 with the PE 150 method.

Trimmomatic (version 0.36) was used to filter out low-quality reads [44]. Clean reads were mapped to the ZmZFP69 genome by Bwa (version 0.7.15) [45]. Samtools (version 1.3.1) was used to remove potential PCR duplicates [46]. MACS2 software (version 2.1.1.20160309) was used to call peaks by default parameters (bandwidth, 300 bp; model fold, 5, 50; FC,2; q value, 0.001). If the midpoint of a peak was located closest to the TSS of one gene, the peak was assigned to that gene [47]. Promoter regions were defined as 2 kb upstream of the TSS. Peaks were assigned to genes if their midpoint was within 2 kb of the TSS. Genes with peaks in this region were considered potential direct targets of ZmZFP69. HOMER (version 3) was used to predict motif occurrence within peaks with default settings for a maximum motif length of 12 base pairs [48]. ClusterProfiler (http://www.bioconductor.org/packages/release/bioc/html/clusterProfiler.html, accessed on 10 December 2024) in the R package [49] was employed to perform GO [50] (Gene Ontology, http://geneontology.org/) and KEGG [51] (Kyoto Encyclopedia of Genes and Genomes, http://www.genome.jp/kegg/, accessed on 10 December 2024) enrichment analyses. The GO and KEGG enrichment analyses were conducted using a hypergeometric distribution with a q-value cutoff of 0.05 [49].

### 4.3. Luciferase Reporter Assay

The full-length coding regions of *ZmZFP69* and *ZmAOX2* were cloned into the LUC reporter vectors pCAMBIA1300-cLUC and pCAMBIA1300-nLUC, resulting in *ZmZFP69*-cLUC and *ZmAOX2*-nLUC, respectively. Agrobacterium tumefaciens cells harboring these two vectors were co-infiltrated into *N. benthamiana* leaves. After 2 d incubation in planta, the leaves were injected with 1 mM D-Luciferin (50227-1MG; Sigma-Aldrich) before the LUC bioluminescence intensity was analyzed using a Tanon-5200 Chemiluminescent Imaging System (Tanon, Shanghai, China). LUC/REN ratio was normalized to Renilla luciferase activity (pCambia1300-221-Flag as internal control), with lower ratios indicating promoter repression.

### 4.4. InterProDesign Identification of Downstream Interacting Proteins

The identification work of InterProDesign was carried out by Hefei Kejing Biotechnology Co., Ltd. (Hefei, China). By combining generative AI models with structural biology analysis, the binding design is based on the target domain of ZmZFP69 (Zm00001d052815) and the information of hotspot residues. Using the RFdiffusion generative model, starting from the known structure of the bait protein ZmZFP69, protein sequences that may have high interaction potential with it were designed from scratch based on the core interaction region. Subsequently, these designed proteins were screened using the three-dimensional protein structure database of the target species through the Foldseek tool, and natural proteins with similar structures were identified. The Hdock binding energy was calculated, and the AF3 (AlphaFold3) complex was predicted for the top 100 proteins with a Bit score greater than 50 in FoldSeek [52,53]. For the screened candidate proteins, their binding energy with ZmZFP69 was further evaluated through the HDOCK tool. Meanwhile, the confidence level of the overall structure of the protein complex was verified in combination with AlphaFold 3 to comprehensively evaluate the interaction stability of the candidate proteins. Subsequently, the data screened out by Hdock were transferred to AlphaFold3 to calculate the pTM + ipTM values. The sequences of the target proteins ZmZFP69 and ZmBG6 were obtained from Uniport and subsequently modeled and docked using AlphaFold3. AlphaFold3 employs an end-to-end transformer-based architecture, leveraging both evolutionary multiple sequence alignments (MSAs) and physical constraints to predict highly accurate three-dimensional protein structures. The docking results were analyzed and visualized using PyMOL 2.6.1, a widely used molecular visualization tool. The final complex structures were rendered in high-resolution three-dimensional graphics, highlighting key interactions [54,55].

### 4.5. BiFC and Subcellular Localization Assays

The full-length coding regions of *ZmZFP69* and *ZmBG6* were individually cloned to the Gateway entry vector pQBV3 before being transferred to their destination vectors, including pEarleyGate101-YFP, pEarlyGate201-nYFP, and pEarlyGate201-cYFP, with the LR clonase kit (Invitrogen). Agrobacterium tumefaciens cells harboring these vectors were infiltrated into *N. benthamiana* leaves. Following a period of 2 d incubation in the dark, the fluorescence signal was analyzed using a laser scanning confocal microscope (Zeiss LSM 880; Carl Zeiss, Jena, Germany). A vector expressing the histone H2B-mCherry, pPTN828, was co-infiltrated with these vectors, allowing for nuclear-specific visualization [56]. All the images were processed using the ZEN BLUE v.2.1 software (Zeiss Microsystems; Carl Zeiss, Jena, Germany).

## Figures and Tables

**Table 1:** Putative target genes of *ZmZFP69* identified by RNA-seq/DAP-seq integration.

No.	Gene ID	Name	Pathway	Notification
1	*Zm00001d034298*	*ZmPIF4*	Plant hormone signal transduction	phytochrome-interacting factor 4
2	*Zm00001d029577*	*ZmZH3*	Plant hormone signal transduction	INDOLE-3-ACETIC ACID-AMIDO SYNTHETASE GH3.2-RELATED
3	*Zm00001d016294*	*ZmPYL2*	MAPK signaling pathway-plant	ABSCISIC ACID RECEPTOR PYL2-RELATED
4	*Zm00001d013098*	*ZmAOX2*	Indole acetaldehyde oxidase	Indoleacetal dehyde oxidase

**Table 2:** Prediction information table for *ZmZFP69* interaction proteins.

No.	Protein Name	Hdock Docking Score (kcal/mol)	Protein Description	Gene ID
1	A0A1D6PKZ7	−356.74	PTHR33322:SF4-BAG FAMILY MOLECULAR CHAPERONE REGULATOR 6	*Zm00001d048489* (ZmBG6)
2	K7UPU9	−349.24	PF00931//PF01582-NB-ARC domain (NB-ARC)//TIR domain (TIR)	*Zm00001d053244* (ZmTIR)
3	A0A1D6K803	−342.77	2.7.11.1-Non-specific serine/threonine protein kinase/Threonine-specific protein kinase	*Zm00001d029829* (ZmTSP)
4	B6TCP0	−342.09	PTHR13856//PTHR13856:SF81-VHS DOMAIN CONTAINING PROTEIN FAMILY//ENTH/VHS/GAT FAMILY PROTEI	*Zm00001d035029* (ZmGAT)
5	B6UIN4	−320.92	Tetratricopeptide repeat (TPR)-like superfamily protein	*Zm00001d052177* (ZmTPR)
6	A0A1D6KFQ1	−304.85	KOG0978-E3 ubiquitin ligase involved in syntaxin degradation	*Zm00001d031005* (ZmUBL)

## Data Availability

The RNA-Seq data of this study have been uploaded to the GEO (Gene Expression Omnibus) database. https://www.ncbi.nlm.nih.gov/geo/query/acc.cgi?acc=GSE299259 (accessed on 7 June 2025).

## Supplementary Materials

The following supporting information can be downloaded at https://www.mdpi.com/article/10.3390/plants14142114/s1.
